# Heterogeneous nuclear ribonucleoproteins A1 and A2 modulate expression of Tid1 isoforms and EGFR signaling in non-small cell lung cancer

**DOI:** 10.18632/oncotarget.7606

**Published:** 2016-02-23

**Authors:** Chi-Yuan Chen, Chia-Ing Jan, Wen-Chieh Pi, Wen-Lung Wang, Pan-Chyr Yang, Tong-Hong Wang, Rotem Karni, Tzu-Chien V. Wang

**Affiliations:** ^1^ Graduate Institute of Health Industry Technology and Research Center for Industry of Human Ecology, College of Human Ecology, Chang Gung University of Science and Technology, Kwei-San, Tao-Yuan 333, Taiwan; ^2^ Department of Pathology, China Medical University and Hospital, Taichung, Taiwan 404, Taiwan; ^3^ Department of Pathology, China Medical University and Beigang Hospital, Yunlin, Taiwan 651, Taiwan; ^4^ Department of Molecular and Cellular Biology, College of Medicine, Chang Gung University, Kwei-San, Tao-Yuan 333, Taiwan; ^5^ Department of Otolaryngology, Kaohsiung Chang Gung Memorial Hospital, Kaohsiung City 833, Taiwan; ^6^ Department of Internal Medicine, College of Medicine, National Taiwan University, Taipei 100, Taiwan; ^7^ Tissue Bank, Chang Gung Memorial Hospital, Tao-Yuan 333, Taiwan; ^8^ The Institute for Medical Research Israel-Canada, The Hebrew University-Hadassah Medical School, Ein Karem, 91120, Jerusalem, Israel

**Keywords:** hnRNP A1, hnRNP A2, Tid1, EGFR, NSCLC

## Abstract

The Tid1 protein is a DnaJ co-chaperone that has two alternative splicing isoforms: Tid1 long form (Tid1-L) and Tid1 short form (Tid1-S). Recent studies have shown that Tid1-L functions as a tumor suppressor by decreasing EGFR signaling in various cancers, including head and neck cancer and non-small cell lung cancer (NSCLC). However, the molecular mechanism responsible for regulating the alternative splicing of Tid1 is not yet known. Two splicing factors, heterogeneous nuclear ribonucleoproteins (hnRNP) A1 and A2, participate in alternative splicing and are known to be overexpressed in lung cancers. In this work, we examined if hnRNP A1 and A2 could regulate the alternative splicing of Tid1 to modulate tumorigenesis in NSCLC. We report that RNAi-mediated depletion of both hnRNP A1/A2 (but not single depletion of either) increased Tid1-L expression, inhibited cell proliferation and attenuated EGFR signaling. Analyses of the expression levels of hnRNP A1, hnRNP A2, EGFR and Tid1-L in NSCLC tissues revealed that hnRNP A1 and A2 are positively correlated with EGFR, but negatively correlated with Tid1-L. NSCLC patients with high-level expression of hnRNP A1, hnRNP A2 and EGFR combined with low-level expression of Tid1-L were associated with poor overall survival. Taken together, our results suggest that hnRNP A1 or A2 are both capable of facilitating the alternative splicing of exon 11 in the Tid1 pre-mRNA, thereby suppressing the expression of Tid1-L and allowing EGFR-related signaling to facilitate NSCLC tumorigenesis.

## INTRODUCTION

Tid1/Dnaja3, a member of the DnaJ co-chaperone family, has been shown to act as a tumor suppressor in head and neck cancer and non-small cell lung cancer (NSCLC) [1, 2]. Human Tid1 spans approximately 34 kb and is composed of 12 exons separated by 11 introns. Comparison of the cDNA sequences of Tid1 to the genomic structure of heteronuclear RNA (hnRNA) strongly suggests that alternative splicing generates the two Tid1 isoforms from a single hnRNA species [3]. The two isoforms, Tid1 long form (Tid1-L) and Tid1 short form (Tid1-S) are expressed in most cells [3]. Tid1-L corresponds to the long form of the Tid1 mRNA and incorporates all 12 exons within the mature transcript, whereas the short form, Tid1-S, is generated by the splicing of exon 10 to exon 12 [3].

The relative expression levels and functions of Tid1-L and Tid1-S vary across different cell types, and Tid1-L has been shown to undergo transcriptional and translational down-regulation in NSCLC cell lines and patients [2]. Previous studies have shown that Tid1-L decreases the stability of HIF-1α and down-regulates VEGF to modulate the neovascularization of endothelial cells *in vitro* and *in vivo* [4]. Tid1-L has also been reported to suppress transformation in human cancer cells, including the A549 lung cancer cell line [5], and it reportedly binds with a number of cell signaling molecules, such as von Hippel-Lindau protein (pVHL) [4], HTLV-1 tax [6], and HSP70 [7, 8]. Tid1-S has been reported to enhance HGF-mediated migration in human renal cell carcinoma cell line 786-0 [9], and overexpression of Tid1-S partially rescued colorectal cancer cells from apoptosis mediated by the caspase-cleaved adenomatous polyposis cell tumor suppressor [10]. In the U2OS osteosarcoma cell line, overexpression of Tid1-L or Tid1-S has opposing effects on apoptosis induced by the DNA-damaging agent, mitomycin C [8]. These observations suggest that the two isoforms of Tid1 play important roles in various cellular processes, including immune responses, apoptosis, angiogenesis, senescence, and development. However, the molecular basis responsible for regulating the alternative splicing of Tid1 remains largely unknown.

The heterogeneous nuclear ribonucleoproteins (hnRNPs) constitute a large family of proteins that associate with nascent pre-mRNAs and package them into hnRNP particles. The A/B hnRNPs, which comprise the most abundant hnRNP subfamily in the nucleus of proliferating cells, are essential components of the spliceosome and are involved in both constitutive and alternative splicing [11]. HnRNP A1 and A2 are among the few hnRNP proteins that are assembled into spliceosomes at all major splicing stages. Recent studies have indicated that hnRNP A1 and A2 modulate alternative splicing of the glycolytic PKM2 enzyme in cancer cells, suggesting that these hnRNPs may be involved in regulating tumor metabolism [12, 13]. Up-regulation of hnRNP A1 and (more profoundly) hnRNP A2 in hepatocellular carcinoma triggers an alternative splicing switch that down-regulates a dominant-negative isoform of A-Raf, leading to activation of the Raf-MEK-ERK pathway [14]. The functions of hnRNP A1 and A2 in the alternative splicing of these oncogenes and tumor-related genes may explain the frequent dysregulation of these hnRNPs in different types of cancer [15].

SELEX-based studies were previously used to identify the consensus binding sequences of hnRNP A1 and A2 (UAGGGA and UAGGGU, respectively) in introns 10 and 11 of the Tid1 pre-mRNA [16, 17]. Since hnRNP A1 and A2 are reportedly overexpressed in lung cancer [18, 19], and depletion of hnRNP A2 reduces AKT activity and Slug expression in NSCLC cell lines [20], we postulated that hnRNP A1 and A2 may regulate the alternative splicing of Tid1 to modulate tumorigenesis in human NSCLC. In this study, we examined how the expression levels hnRNP A1 and A2 affect the alternative splicing of Tid1 and the EGFR signaling pathway in NSCLC.

## RESULTS

### Tid1 isoform expression in cells depleted of hnRNP A1 and/or A2

**Figure 1:**
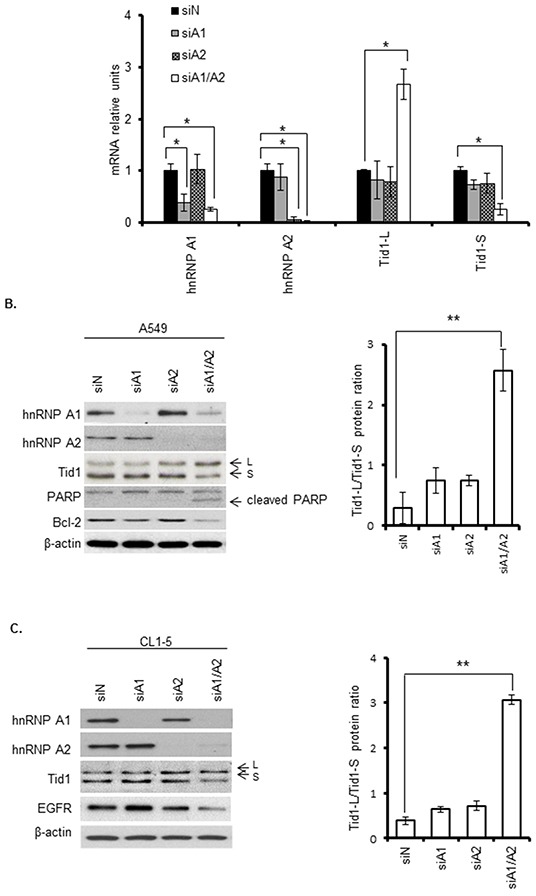
Effects of hnRNP A1 and/or A2 depletion on the expression levels of Tid1 isoforms Cells were transfected with specific siRNAs targeting hnRNP A1 (siA1), hnRNP A2 (siA2), both hnRNP A1 and A2 (siA1/A2), or non-target sequences (siN, control). After transfection for 72 h, cells were harvested and the expression levels of the target genes and Tid1 splicing variants were analyzed. **A.** Quantitative (q)RT-PCR analysis of the mRNA expression levels of the Tid1 variants in A549 cells treated as described above. **B** and **C.** Western blot analyses of the relevant protein levels in A549 cells (B) and CL1-5 cells (C). Induction of apoptosis was analyzed by detecting the PARP cleavage product (cl-PARP) and Bcl-2 expression in transfected cells. The Tid1-L and Tid1-S splicing variants are indicated with arrows and labeled as “L” and “S,” respectively. The levels of Tid1-L and Tid1-S were quantified by densitometry, and the Tid1-L/Tid1-S ratios from five and three independent experiments are shown in the right panels of (B) and (C), respectively. β-Actin served as the loading control. Symbols: *p < 0.05, **p < 0.01 based on the Student's t-test.

To test our hypothesis that hnRNP A1 and/or A2 may be involved in the alternative splicing of Tid1 isoforms in NSCLC, we transfected A549 cells with siRNAs targeting hnRNP A1 and/or A2, and assessed the expression levels of the Tid1 isoforms using qRT-PCR and Western blot analyses. As shown in Figure 1, treatment of cells with siRNAs targeting hnRNP A1, hnRNP A2, or both (hnRNP A1/A2) effectively reduced the relevant mRNA (Figure 1A) and protein (Figure 1B) levels. Single depletion of hnRNP A1 or A2 alone did not appear to affect the relative mRNA expression levels of Tid1-L or Tid1-S; however, simultaneous suppression of hnRNP A1 and A2 (hnRNP A1/A2) increased the mRNA expression level of Tid1-L while reducing that of Tid1-S (Figure 1A). At the protein level, single depletion of hnRNP A2 alone appeared to slightly increase the relative ratio of Tid1-L/Tid1-S (Figure 1B), while hnRNP A1/A2 double depletion greatly increased the relative expression ratio of Tid1-L/Tid1-S (Figure 1B). Similar results were obtained with CL1-5 cells (another NSCLC cell line; Figure 1C), indicating that simultaneous suppression of hnRNP A1/A2 (but not single suppression of either alone) facilitates the expression of Tid1-L.

### Cell proliferation, anchorage-independent growth, apoptosis, and EGFR signaling in cells depleted of hnRNP A1 and/or A2

**Figure 2:**
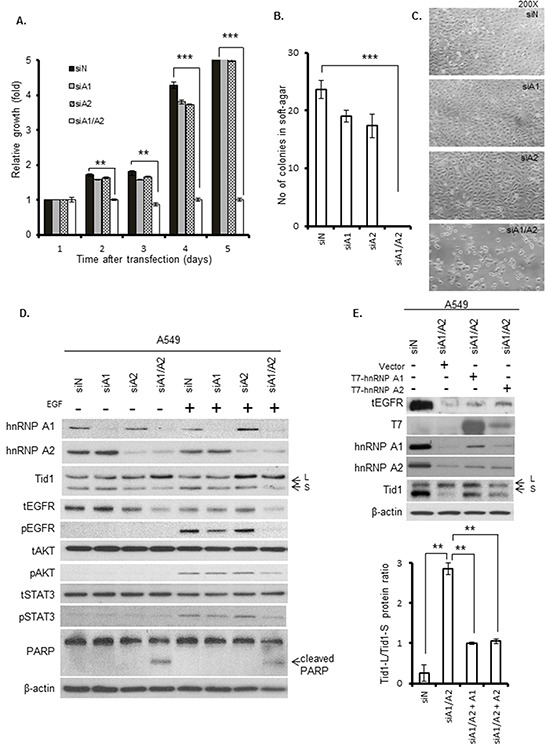
Effects of hnRNP A1 and A2 depletion on cell proliferation, anchorage-independent growth, apoptosis, and EGFR signaling A549 cells were transfected with siA1, siA2, siA1/A2, or control siN for 72 h, and then cultured for the indicated durations in the absence of transfection reagents. **A.** Cell proliferation was determined by MTT assay. **B.** Anchorage-independent growth was determined by plating the transfected cells in soft agar, culturing the cells for 2 weeks, and scoring the number of colonies. Symbol: ***, *p* < 0.001 based on the Student's *t*-test. **C.** Microscopic morphology of cells after transfection for 72 h. **D.** Immunoblotting of cell lysates with the indicated primary antibodies. The transfected cells were cultured in the absence of serum for 24 h, and then incubated in the presence or absence of 50 ng/mL EGF at 37°C for 15 min. The total and phosphorylated forms of EGFR, STAT3 and AKT are indicated by the prefixes “t” and “p”, respectively. The Tid1-L and Tid1-S splicing variants are indicated with arrows labeled “L” and “S,” respectively. β-Actin was used as an internal control. **E.** A549 cells were transfected with siA1/A2, or control siN. After 24 h, the transfection reagents were removed and the cells were cultivated in regular medium for 4 h. The hnRNP A1/A2 depleted cells were then transfected with plasmids expressing T7-tagged hnRNP A1 (T7-hnRNP A1), hnRNP A2 (T7-hnRNP A2) or the empty vector (Vector). After 24 h, the transfection reagents were removed and the transfected cells were cultured for additional 24 h in regular medium before being analyzed for the expression of proteins by Western blot (top panel). The levels of Tid1-L and Tid1-S were quantified by densitometry, and the Tid1-L/Tid1-S ratios from three independent experiments are shown in bottom panel.

To evaluate whether the suppression of hnRNP A1 and/or A2 could affect the tumorigenesis of NSCLC, we depleted hnRNP A1 or/and A2 in A549 cells and examined cell proliferation, anchorage-independent growth, apoptosis, and EGFR signaling. Single depletion of hnRNP A1 or A2 alone did not appear to affect cell proliferation (Figure 2A and Supplementary Figure S1) or anchorage-independent growth (Figure 2B), but hnRNP A1/A2 double depletion completely inhibited these processes. Single depletion of hnRNP A1 or A2 alone also did not appear to induce apoptosis (Figure 1B) or affect cell morphology (Figure 2C), but hnRNP A1/A2 double depletion was associated with cytoplasmic condensation and the formation of apoptotic bodies (Figure 2C), as well as reduced expression of Bcl-2 and increased cleavage of PARP (Figure 1B). These findings indicate that the double depletion was associated with the induction of apoptosis.

To test whether depletion of hnRNP A1 and/or A2 could affect EGFR-dependent signaling in NSCLC, A549 cells were transfected with siRNAs against hnRNP A1, hnRNP A2, or both (hnRNP A1/A2) for 72 h. The transfected cells were serum-starved for 24 h, and then stimulated with 50 ng/mL of EGF for 15 minutes. We found that serum starvation reduced the level of hnRNP A1 and increased the ratio of Tid1-L/Tid1-S (Supplementary Figure S2). In serum-starved cells, EGF stimulation activated EGFR signaling via phosphorylation of EGFR and its downstream substrates, AKT and STAT3 (Supplementary Figure S2). Depletion of hnRNP A1 or A2 alone had no effect on the EGF-stimulated phosphorylation of EGFR, AKT or STAT3 (Figure 2D), whereas hnRNP A1/A2 double depletion reduced the total level of EGFR and inhibited the phosphorylation of EGFR, AKT and STAT3 (Figure 2D). Consistent with this, hnRNP A1/A2 double depletion also increased the expression of Tid1-L and reduced EGFR in CL1-5 cells (Figure 1C).

To determine if the expression of hnRNP A1 or hnRNP A2 could increase the expression of Tid1-S and EGFR signaling, the hnRNP A1/A2 double-depleted cells were transfected with plasmids expressing T7-tagged hnRNP A1 or hnRNP A2 and the effects of ectopic expression on the levels of Tid1 isoforms and tEGFR were examined. As shown in Figure 2E, ectopic expression of T7-tagged hnRNP A1 or A2 increased the expression level of Tid1-S and partially restored the Tid1-L/Tid1-S ratio towards that of the siN transfected control cells. Similarly, the tEGFR level was also partially restored by the ectopic expression of either hnRNP A1 or A2. Taken together, these results are consistent with the idea that hnRNP A1 and A2 can regulate the alternative splicing of Tid1, thereby affecting EGFR-dependent signaling in NSCLC cells.

### Expression levels of hnRNP A1, hnRNP A2, EGFR, and Tid1 isoforms in NSCLC tissues

**Figure 3:**
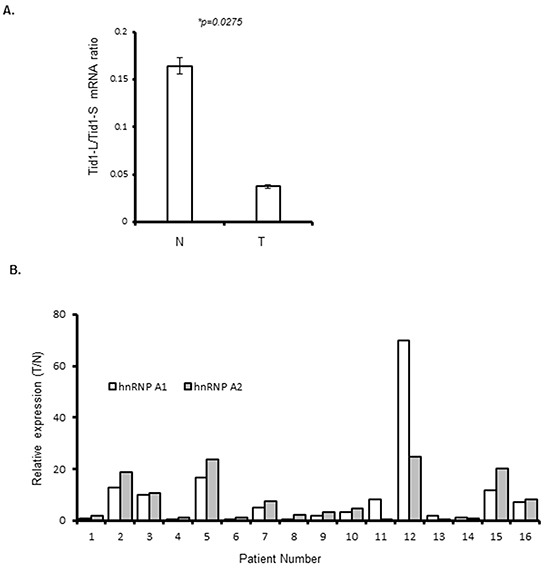
Quantitative (q)RT-PCR analysis of Tid1 isoforms, hnRNP A1, and hnRNP A2 in NSCLC tissues **A.** Relative Tid1-L/Tid1-S mRNA ratios in non-cancerous tissues (N) and tumor tissues (T) from NSCLC patients of Group 1 (n = 16). The mRNA expression levels of Tid1-L and Tid1-S were determined by qRT-PCR and normalized with respect to GAPDH, and the ratio of Tid1-L/Tid1-S was determined for each sample. Symbols: *p < 0.05 by Student's *t*-test; and bars represent means ± SD from three experiments. **B.** The mRNA expression levels of hnRNP A1 and A2 were determined by qRT-PCR in N and T samples from the 16 NSCLC patients of Group 1, and normalized with respect to GAPDH. The histograms show the relative expression levels of hnRNP A1 and A2 in the tumor and normal tissues (T/N) of each patient.

To assess whether the relative expression levels of the Tid1 isoforms may be altered in NSCLC, we examined these levels in NSCLC tumors (T) and adjacent normal lung tissues (N) from 16 patients. The mRNA expression levels of Tid1-L and Tid1-S were quantitated using real-time RT-PCR, and the Tid1-L/Tid1-S ratios were compared between human lung tumor samples and adjacent normal lung tissues. As shown in Figure 3A, the relative ratios of Tid1-L/Tid1-S were significantly lower in the tumor tissues compared to the adjacent normal tissues, indicating that alternative splicing favors the Tid1-S mRNA in NSCLC tumors.

To examine whether the bias of alternative splicing in favor of Tid1-S could be correlated with the expression levels of hnRNP A1 and/or A2 in NSCLC, we used qRT-PCR to compare their relative mRNA expression levels in T and N tissues from the 16 NSCLC patients. The ratio of relative expression (T/N) was arbitrarily considered as significant when it is higher than 2. According to this criterion, higher expression for both hnRNP A1 and A2 was observed in nine patients, higher for hnRNP A1 alone was observed in one patient, and higher for hnRNP A2 alone was observed in one patient (Figure 3B).

**Figure 4:**
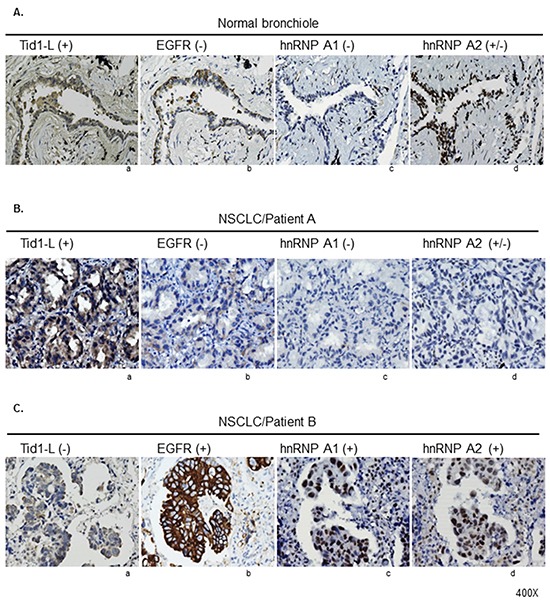
Immunohistochemical (IHC) staining of Tid1-L, hnRNP A1, hnRNP A2, and EGFR in NSCLC tissues The expression levels of Tid1-L, EGFR, hnRNP A1, and hnRNP A2 in normal bronchiole **A.** and tumor tissues from two NSCLC patients **B** and **C.** were determined by IHC staining. Representative examples of positive staining (+), weakly positive staining (+/−), and negative staining (−) are indicated.

To further evaluate the relationship among hnRNP A1, hnRNP A2, and Tid1-L *in vivo*, the relative expression levels of these proteins were examined by IHC in paired T and N tissues from 49 patients with NSCLC (Group 2). The clinical features of these NSCLC patients are summarized in Supplementary Table S1. We also analyzed epidermal growth factor receptor (EGFR) signaling, which is a major driver of lung adenocarcinoma and has been shown to be negatively regulated by Tid1-L [2]. Serial sections of each sample were stained with antibodies against hnRNP A1, hnRNP A2, Tid1-L, and EGFR. A computerized image analysis system was used for IHC scoring, as described previously [2]. Figure 4 presents representative staining patterns for normal bronchiole (Figure 4A) and tumor samples from two NSCLC patients (Figures 4B and 4C). In the normal bronchiole (Figure 4A), we observed positive staining (+) for Tid1-L, negative staining (−) for EGFR and hnRNP A1, and weakly positive staining (+/−) for hnRNP A2. In the tumor sample of patient A (Figure 4B), we observed positive staining for Tid1-L, negative staining (−) for EGFR and hnRANP A1, and weakly positive staining (+/−) for hnRNP A2. In the tumor sample of patient B (Figure 4C), we observed negative staining (−) for Tid1-L and positive staining (+) for hnRNP A1, hnRNP A2, and EGFR. In total, 13/49, 31/49, 20/49, and 34/49 of the tumor samples stained positively for Tid1-L, EGFR, hnRNP A1, and hnRNP A2, respectively. Negative (−) or weakly positive staining (+/−) was observed in 36/49, 18/49, 29/49, and 15/49 of the tumor samples for Tid1-L, EGFR, hnRNP A1, and hnRNP A2, respectively.

The correlations between the histologically detected expressions of Tid1-L, hnRNP A1, and hnRNP A2 are summarized in Table 1. Low-level expression of Tid1-L was strongly correlated with high-level expressions of hnRNP A1 or A2 (Table 1; *p* = 0.045 and 0.034, respectively). Moreover, low-level expression of Tid1-L was associated with high-level expression of hnRNP A1 or A2 (Table 2, *p* = 0.034), whereas high-level expression of hnRNP A1 or A2 was strongly correlated with high-level expression of EGFR (Table 2, *p* = 0.039).

**Table 1:** Correlations between the expression level of Tid1-L and those of hnRNP A1 or hnRNP A2 in the tumors of 49 NSCLC patients (Group 2)

Characteristic	Tid1-L		*P* value^c^
	Low^a^ N=36)	High^b^ (N=13)	
HnRNP A1			
Low^a^	17	12	0.045*
High^b^	19	1	
HnRNP A2			
Low^a^	8	7	0.034*
High^b^	28	6	

a Low: NSCLC tumors showing negative or weak staining for Tid1-L, hnRNP A1 or hnRNP A2 (scores of 0 or 1).

b High: NSCLC tumors showing intense staining for Tid1-L, hnRNP A1 or hnRNP A (score of 2).

c Analyzed by Chi-square test.

**Table 2:** Correlations between the expression level of hnRNP A1/A2 and those of Tid1-L or EGFR in tumor tissues from 49 NSCLC patients (Group 2)

Characteristic	HnRNP A1/A2		*P* value^e^
	Low^a^ (N=15)	High^b^ (N=34)	
Tid1-L			
Low^c^	8	28	0.034*
High^d^	7	6	
EGFR			
Low^c^	10	8	0.039*
High^d^	5	26	

a Low: NSCLC tumors showing negative or weak staining for both hnRNP A1 and hnRNP A2 (scores of 0 or 1).

b High: NSCLC tumors showing intense staining for hnRNP A1, hnRNP A2, or both (score of 2 for one or both).

c Low: NSCLC tumors showing negative or weak staining for Tid1-L or EGFR (scores of 0 or 1).

d High: NSCLC tumors showing intense staining for Tid1-L or EGFR (score of 2).

e Analyzed by Chi-square test.

### Correlation of the low-Tid1-L/high-hnRNP A1/A2 expression signature with poor overall survival of NSCLC patients

**Figure 5:**
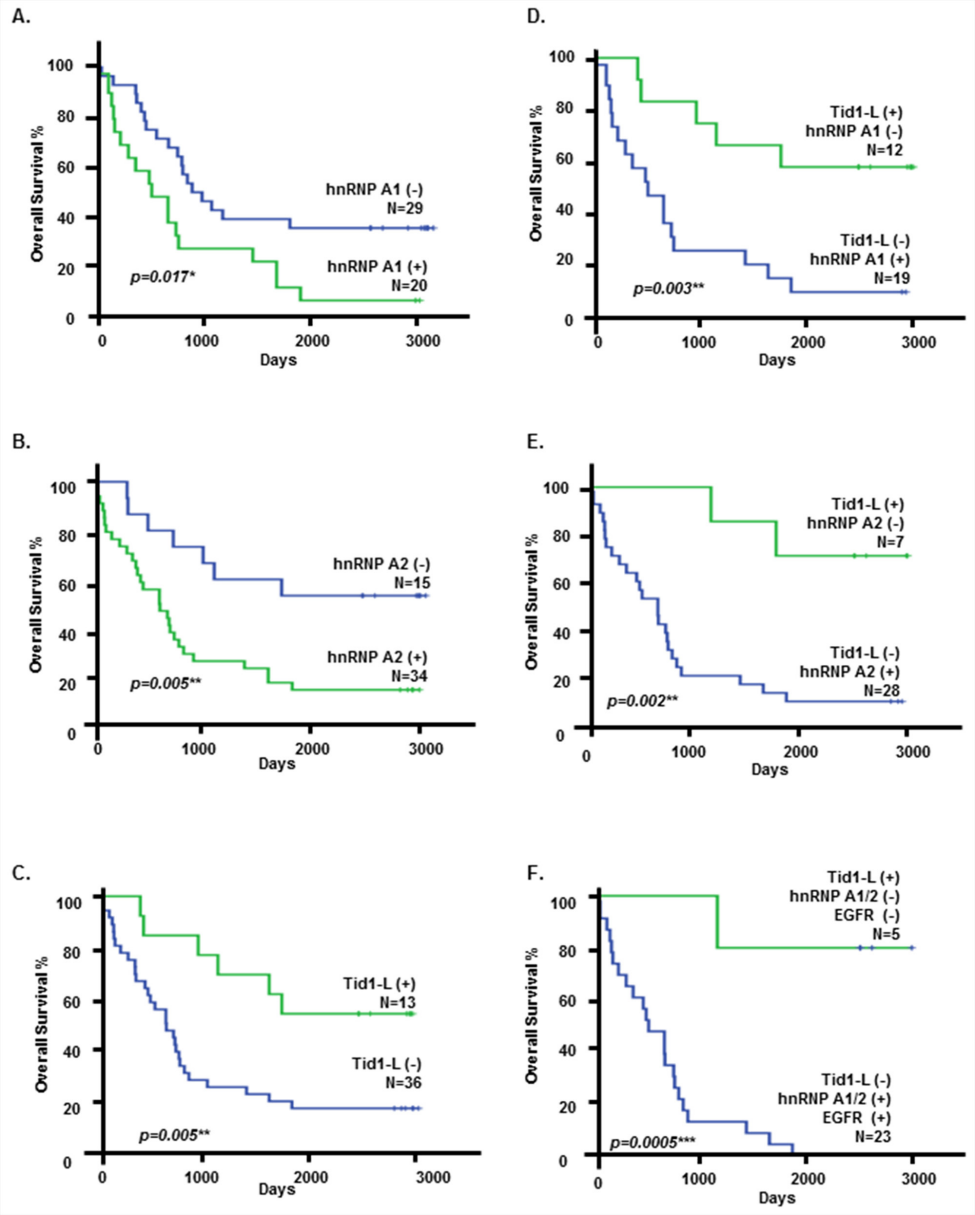
Kaplan-Meier analysis of overall survival in NSCLC patients Kaplan-Meier analysis of overall survival of NSCLC patients (Group 2) was stratified according to the hnRNP A1 score **A.**; the hnRNP A2 score **B.**; the Tid1-L score **C.**; grouped as Tid1-L(+)/hnRNP A1(-) or Tid1-L(-)/hnRNP A1(+) **D.**; grouped as Tid1-L(+)/hnRNP A2(-) or Tid1-L(-)/hnRNP A2(+) **E.**; and grouped as Tid1-L(+)/hnRNP A1/2(-)/EGFR(-) or Tid1-L(-)/hnRNP A1/A2(+)/EGFR(+) **F.**

To enable multivariate analysis of the expression levels of Tid1-L, hnRNP A1, hnRNP A2, and EGFR and their impacts on survival, the scores for Tid1-L, hnRNP A1, hnRNP A2, and EGFR were dichotomized into positive versus negative expression; positive corresponded to a score of 2 and negative to scores of 0 or 1 for Tid1-L, hnRNP A1, and hnRNP A2, while positive corresponded to scores of 2 or 3 and negative to scores of 0 or 1 for EGFR. Kaplan-Meier survival analysis revealed that patients with positive expression of hnRNP A1 or hnRNP A2 had poorer overall survival than those with negative expression of hnRNP A1 or hnRNP A2 (Figure 5A and 5B; *p* = 0.017 and 0.005, respectively). Likewise, patients with positive expression of Tid1-L had better overall survival compared with those with negative expression of Tid1-L (Figure 5C; *p* = 0.005). To gain further insights into the effects of Tid1-L and hnRNP A1 or A2 expression on patient survival, we conducted survival analyses for patients who were Tid1-L (+) but hnRNP A1 (-) or hnRNP A2 (-), and also for patients who were Tid1-L (-) but hnRNP A1 (+) or hnRNP A2 (+). As shown in Figures 5D and 5E, patients with Tid1-L (+)/hnRNP A1 (-) or Tid1-L (+)/hnRNP A2 (-) had better overall survival rates than those with Tid1-L (-)/hnRNP A1 (+) or Tid1-L (-)/hnRNP A2 (+). As shown in Figure 5F, patients with Tid1-L (+)/hnRNP A1/A2 (−)/EGFR (-) had better overall survival than those with Tid1-L (−)/hnRNP A1/A2 (+)/EGFR (+) (*p* = 0.0005).

## DISCUSSION

The Tid1 isoforms play important physiological roles in a variety of cellular processes, including immune responses, apoptosis, senescence, and development. Intron 10 and 11 of the Tid1 pre-mRNA contain the consensus binding sequences for hnRNP A1 and A2 [UAGGG (A/U)] [16, 17], prompting us to speculate that this gene could be regulated by hnRNP A1 and/or A2. Here, we show that the simultaneous depletion of both hnRNP A1 and A2 in human NSCLC cells increases Tid1-L expression, induces apoptosis, inhibits cell proliferation and decreases EGFR signaling in these cells (Figures 1 and 2). Single depletion of hnRNP A1 or A2 alone did not appear to affect cell proliferation or the relative mRNA expression levels of Tid1-L or Tid1-S (Figure 1), suggesting that the expression of either hnRNP A1 or A2 is sufficient to facilitate the expression of Tid1-S (i.e., the exclusion of exon 11 of the Tid1-encoding gene).

Since increased expression of Tid1-L has been shown to inhibit tumorigenesis in NSCLC [2] and our present cell culture studies indicate that hnRNP A1 and A2 modulate the expression of Tid1 isoforms, we examined whether Tid1-L expression was correlated with the expression levels of these hnRNPs in NSCLC tissues. As shown in Figure 4, Tid1-L is expressed in normal bronchiole of human lung tissues. The relative ratios of Tid1-L/Tid1-S were significantly lower in the tumor tissues compared to the adjacent normal tissues (Figure 3), which is in agreement with the previous finding that Tid1-L was reduced in the majority of NSCLC tumor samples [2]. Correlation analysis of our histological results indicated that: 1) low-level expression of Tid1-L was strongly correlated with high-level expressions of hnRNP A1 or hnRNP A2 (Table 1; *p* = 0.045 and 0.034, respectively); and 2) high-level expressions of hnRNP A1 or/and hnRNP A2 were correlated with low-level expression of Tid1-L (Table 2, *p* = 0.034) but high-level expression of EGFR (Table 2, *p* = 0.039). These results are consistent with the idea that expression of either hnRNP A1 or hnRNP A2 can facilitate the alternative splicing of exon 11 in the Tid1 pre-mRNA, thereby suppressing the expression of Tid1-L and facilitating NSCLC tumorigenesis.

Interestingly, survival analysis indicated that the overall survival of patients was negatively correlated with the expression levels of hnRNP A1 and A2 (i.e., high levels were associated with poor survival) but positively correlated with the expression level of Tid1-L (i.e., low expression was correlated with poor survival) (Figure 5). These clinical correlations further suggest that the expression levels of hnRNP A1/A2, Tid1 isoforms and EGFR could be useful biomarkers for the management of NSCLC patients.

Several lines of evidence indicate that hnRNP A1 and A2, play oncogenic roles [21–23]. For example, overexpression of hnRNP A1 and A2 proteins has been reported in several cancers, including lung cancer [19, 24–26]. Up-regulation of hnRNP A1 and A2 in hepatocellular carcinoma was shown to trigger an alternative splicing switch that down-regulates a dominant-negative isoform of A-Raf [14]. RNA interference (RNAi)-based loss-of-function assays revealed that depletion of Sam68 and hnRNP A1 favors the expression of the Bcl-xL isoform [27]. In addition, hnRNP A1 and A2 were shown to modulate alternative splicing of the glycolytic PKM2 enzyme in cancer cells, suggesting that these hnRNPs contribute to regulating tumor metabolism [12, 13]. Therefore, our novel finding that hnRNP A1 and A2 function in the alternative splicing of the tumor suppressor, Tid1, provides another example of splicing factors that play an oncogenic role [28, 29].

In conclusion, we herein used cell culture studies to show that simultaneous depletion of hnRNP A1 and A2 increases Tid1-L expression. Based on this, we posit that these hnRNPs may be functionally redundant in promoting the alternative splicing of exon 11 in the Tid1 pre-mRNA. Consistent with this, our histological analyses showed that low-level expression of Tid1-L is strong correlated with high-level expression of hnRNP A1 or A2. Together, our findings suggest a model in which the high-level expression of hnRNP A1 or A2 in NSCLC suppresses Tid1-L expression, thereby allowing EGFR-related signaling to facilitate NSCLC tumorigenesis.

## MATERIALS AND METHODS

### Culture media, antibodies, and oligonucleotides

Culture media and fetal bovine serum were purchased from Life Technologies (Grand Island, NY, USA). Antibodies against cleaved PARP (Asp214), phospho-EGFR (Tyr1068), phospho-STAT3 (Tyr705), phospho-p44/42 ERK (Thr202/Tyr204) and phospho-AKT (Ser473) were purchased from Cell Signaling (Temecula, CA, USA). Antibodies against Tid1 (RS-13), Tid1-L (C-15), hnRNP A1 (F-8), hnRNP A2 (EF-67), STAT3, ERK (K-23), AKT (C-20), EGFR (1005) and Bcl-2 (C-2) were purchased from Santa Cruz Biotechnology, Inc. (Santa Cruz, CA, USA). The antibody against T7 tag was purchased from Novagen. The antibody against β-actin was purchased from Sigma (St. Louis, MO, USA). The antibody against wild-type EGFR (clone EGFR.25), which was used for immunohistochemical staining, was purchased from Leica Microsystems (Darmstadt, Germany). EGF was purchased from Sigma. Oligonucleotides were purchased from Purigo Biotech, Inc. (Taipei, Taiwan), and the locked nucleic acid (LNA) probes were purchased from Roche Applied Science (Indianapolis, IN, USA)

### Patients and tumor specimens

Tissue samples were collected from NSCLC patients who underwent surgical resection at China Medical University and Hospital (Taichung, Taiwan, ROC). Two independent collections of tumors and adjacent normal lung tissues from NSCLC patients were used in this study. The Group 1 samples, which were obtained from 16 patients, were used for RT-PCR studies. The Group 2 samples, which were obtained from 49 patients, were used for immunohistochemically staining. The clinicopathological features of the subjects in Group 2 are summarized in Supplementary Table S1. This study was reviewed and approved by the Institutional Review Board and Ethics Committee of China Medical University and Hospital. Written informed consent was obtained from all patients.

### Cell lines and culture

The A549 human lung cancer cell line was purchased from the American Type Culture Collection (Manassas, VA, USA). The CL1-5 cell line was derived from CL1-0 cells using a Transwell invasion chamber, as previously described [30]. CL1-5 cells were cultivated in RPMI-1640 containing 10% fetal bovine serum, 2 mM sodium pyruvate, 100 U/ml penicillin, and 100 U/ml streptomycin. A549 cells were cultured in Dulbecco's modified Eagle's medium (DMEM) containing 10% fetal bovine serum, 0.5 mM sodium pyruvate, 2.5 mM L-glutamine, 100 U/ml penicillin, and 100 U/ml streptomycin. Cells were grown at 37°C in a humidified incubator containing 5% CO_2_.

### Plasmids

The plasmids expressing T7-tagged hnRNP A1 and hnRNP A2 were constructed by cloning the cDNA of hnRNP A1 and hnRNP A2 [14], respectively, into pWZL Hygro vector. Transfection of plasmid DNA was performed as previously described [1].

### RNA interference (RNAi)

Target genes were down-regulated by RNAi-mediated inhibition of mRNA expression, using a mixture of four siRNAs for each target gene (ON-TARGETplus SMARTpool; Dharmacon) [31]. The siGENOME nontargeting siRNA pool (Dharmacon) was used as the control. The siRNA sequences were submitted to BLAST searches to ensure that each siRNA targeted only one human gene. The four siRNAs targeting the human hnRNP A1 mRNA (GenBank accession no. NM_002136) covered the following: nucleotides 1545-1563 from the start codon (A1-1: CGGAAACCUUGGUGUAGUU), nucleotides 1709–1727 (A1–2: GGGAAUGAAGCUUGUGUAU), nucleotides 746–764 (A1–3: CAACUUCGGUCGUGGA GGA), and nucleotides 1468–1486 (A1–4: UAGAAUU CCUUCAGGGUGA). The four siRNAs targeting the hnRNP A2 mRNA (GenBank accession no. NM_002137) covered the following: nucleotides 825–843 (A2–1: CGGUGGAAAUUUCGGACCA), nucleotides 519–537 (A2–2: GCUGUUUGUUGGCGGAAUU), nucleotides 443-461 (A2–3: GGAGAGUAGUUGAGCCAAA), and nucleotides 863–881 (A2–4: GAGGAGGAUCUGAUG GAUA). Transfection was performed using the Dharmafect 1 transfection reagent (Dharmacon) according to the manufacturer's instructions. In brief, exponentially growing cells were seeded in regular growth medium without antibiotics at 40%–50% confluence. After 24 h, cells were transfected with the siRNA, and then incubated for an additional 72 h.

### RNA isolation and analysis of gene expression by RT-PCR

Total cellular RNA was isolated using the TRIzol reagent (Invitrogen) according to the manufacturer's instructions. Quantitative measurements of transcripts were conducted using real-time PCR (RT-PCR), which was performed on a LightCycler 480 instrument (Roche) using the locked nucleic acid (LNA)-based quantitative real-time PCR (LNA-qPCR) method. The primer pairs and LNA probes were selected from conserved regions using the online ProbeFinder Assay Design Software (Roche Applied Science). The uniqueness of each designed target-specific primer and probe sequence (Supplementary Table S2) was evaluated with a BLAST search. The GAPDH mRNA was amplified for normalization. The quantities of the target mRNAs were analyzed in triplicate, normalized with respect to the GAPDH control, and expressed in relation to a calibrator sample.

### Assays for cell proliferation

Cell proliferation was assayed by using an MTT assay kit (Sigma-Aldrich) and by staining with trypan blue to determine the number of viable cells as previously described [32, 33].

### Anchorage-independent growth

Anchorage-independent growth was assessed as previously described [32]. In brief, 5 × 10^4^ cells were mixed with 2 ml of minimum essential medium (MEM) containing 0.35% agarose (Sigma-Aldrich) and 0.5% fetal bovine serum (FBS), and the resulting mixture was poured onto a 60-mm plastic culture dish that had been pre-solidified with 2 ml of MEM containing 0.7% agarose and 15% FBS. The plates were incubated at 37°C for 2 weeks, and then stained with 0.5% crystal violet. Colonies with diameters ≥ 100 μm were scored. Triplicate samples were examined in each experiment.

### Western blotting

The cell pellets were homogenized in 200 μL lysis buffer (50 mM HEPES, pH 7.5, 150 mM NaCl, 10% glycerol, 1.5 mM MgCl_2_, 1% Triton X-100) supplemented with protease inhibitors, and incubated on ice for 10 min. The samples were centrifuged at 12,000 rpm for 30 min at 4°C, and the protein-containing supernatants were collected. Protein concentrations were determined using the Bio-Rad protein assay, and Western blotting was performed as described previously [1].

### Immunohistochemistry (IHC)

IHC was performed as described previously [34], using antibodies against Tid1-L (C-15), hnRNP A1 (F-8), hnRNP A2 (EF-67) and wild-type EGFR (clone EGFR.25). IHC scoring was performed using a computerized image analysis system, as described previously [2].

### Statistics

Nonparametric analyses, Student's *t*-tests, Chi-square tests, and the Kaplan-Meier method were performed using Prism 5.0 (GraphPad Software) or the Statistical Package for the Social Sciences version 12.0 (SPSS, Inc.). Differences between the variables were considered significant at *p* < 0.05.

## SUPPLEMENTARY FIGURES AND TABLES


