# Supplementary material for: Heterogeneous nuclear ribonucleoproteins A1 and A2 modulate expression of Tid1 isoforms and EGFR signaling in non-small cell lung cancer
Source: Oncotarget. 2016 Feb 23;7(13):16760–72. doi: 10.18632/oncotarget.7606 (PMC4941349; doi:10.18632/oncotarget.7606)
Supplement: Supplementary file 1 [file oncotarget-07-16760-s001.pdf]

SUPPLEMENTARY FIGURES AND TABLES

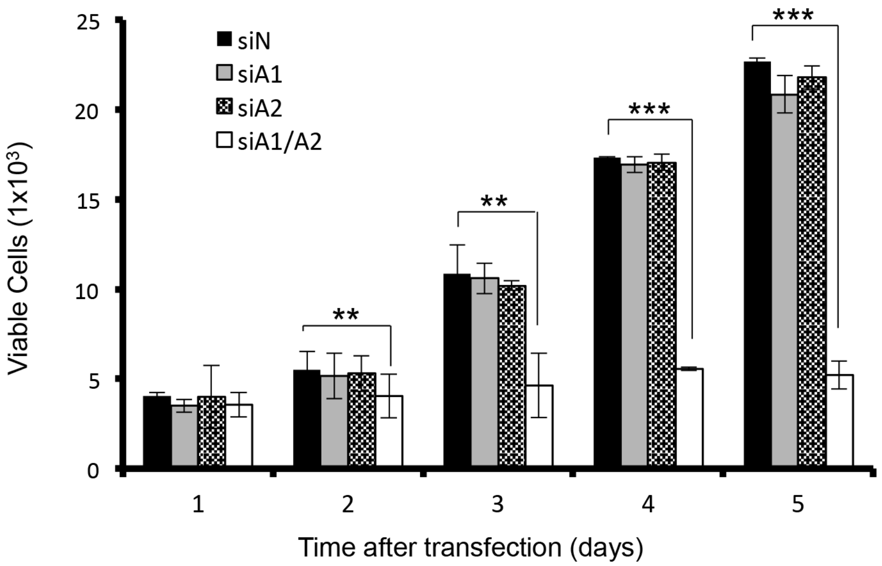

Supplementary Figure S1: Effects of hnRNP A1 and A2 depletion on cell proliferation. A549 cells were transfected with siA1, siA2, siA1/A2, or control siN for 72 h, and then cultured for the indicated durations in the absence of transfection reagents. The number of viable cells at different times was determined by staining with trypan blue and counting with hemocytometer to monitor proliferation. The data are the mean ± SD of three independent experiments. Symbol: **, $p < 0.01$; ***, $p < 0.001$ based on the Student's t-test.

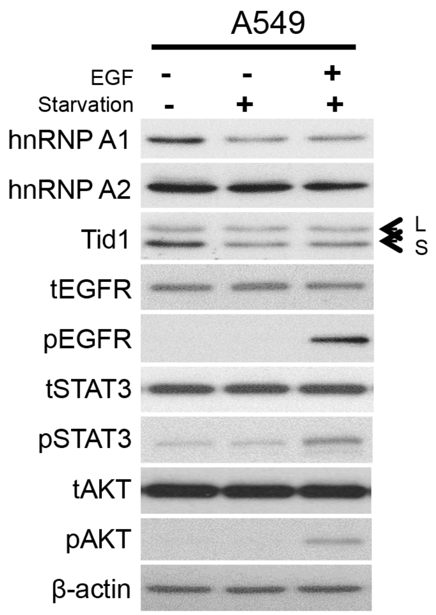

Supplementary Figure S2: Starvation of serum reduces the level of hnRNP A1 and increases the ratio of Tid1-L/Tid1-S in A549 cells. Cells were grown in the presence of absence of serum for 24 h before being harvested for analysis. In the serum-starved cells, the activation of EGFR signaling was examined by treating the starved cells with 50 ng/mL of EGF at 37°C for 15 min. Cell lysates were analyzed by immunoblotting with indicated primary antibodies. The total and phosphorylated forms of EGFR, STAT3 and AKT were indicated by prefix t and p, respectively. The Tid1-L and Tid1-S splicing variants are indicated with arrows L, S, respectively. β-actin was used as internal control. The data shown are representative of 3 independent experiments.

Supplementary Table S1: Characteristics of 49 NSCLC patients (Group 2) with low- or high-level expression of hnRNP A1or hnRNP A2

Characteristic	hnRNP A1		hnRNP A2	
	Low[a] (N=29)	High[b] (N=20)	Low[a] (N=15)	High[b] (N=34)
Age (years)				
≦60	11	7	6	12
>60	18	13	9	22
Gender				
Male	12	9	7	14
Female	17	11	8	20
Stage				
I/II	12	10	8	14
III/IV	17	10	7	20
Grade				
1-2	23	17	11	29
3	6	3	4	5
Lymphovascular invasion				
Absent	17	9	11	15
Present	12	11	4	19

[a]Low: NSCLC showing negative or weak staining for hnRNP A1 or hnRNP A2 (-, scores of 0 or 1).
[b]High: NSCLC with intense staining for hnRNP A1 or hnRNP A2 (+, score of 2).

Supplementary Table S2: Sequences of the utilized primers and LNA probes

Target gene	LNA probe No.	Primer sequence	Product length (nt)
Tid1-L	4	cgtcaccctcaccagctc	100
		tttggaaaggaatccctcct	
Tid1-S	37	cgtcaccctcaccagctc	102
		tctggaatcctcccgtctct	
hnRNP A1	37	ggaagtggtggacagggtta	85
		ctccgcctccgttgttatag	
hnRNP A2	10	tttggggatggctataatgg	71
		ccataaccggggctacct	
GAPDH	60	cactaggcgctcactgttctc	103
		gcccaatacgaccaaatcc	
